# MFG-E8 mediates arterial aging by promoting the proinflammatory phenotype of vascular smooth muscle cells

**DOI:** 10.1186/s12929-019-0559-0

**Published:** 2019-08-30

**Authors:** Hou-Yu Chiang, Pao-Hsien Chu, Ting-Hein Lee

**Affiliations:** 1 Department of Anatomy, College of Medicine, Chang Gung University, 259 Wenhua 1st Rd., Guishan Dist, Taoyuan City, 33302 Taiwan; 2 Graduate Institute of Biomedical Sciences, College of Medicine, Chang Gung University, Taoyuan, Taiwan; 3 Division of Cardiology, Department of Internal Medicine, Chang Gung Memorial Hospital, Linkou, Taiwan; 4 College of Medicine, Chang Gung University, Taoyuan, Taiwan

**Keywords:** Arterial aging, MFG-E8, NF-κB, Cell adhesion molecules, Vascular smooth muscle cells

## Abstract

**Background:**

Among older adults, arterial aging is the major factor contributing to increased risk for cardiovascular disease–related morbidity and mortality. The chronic vascular inflammation that accompanies aging causes diffuse intimal–medial thickening of the arterial wall, thus increasing the vulnerability of aged vessels to vascular insults. Milk fat globule–epidermal growth factor 8 (MFG-E8) is a biomarker for aging arteries. This integrin-binding glycoprotein, induced by angiotensin II, facilitates vascular smooth muscle cell (VSMC) proliferation and invasion in aging vasculatures. This study investigated whether MFG-E8 directly mediates the initial inflammatory responses in aged arteries or VSMCs.

**Methods:**

A model of neointimal hyperplasia was induced in the common carotid artery (CCA) of aged mice to exacerbate age-associated vascular remodeling. Recombinant MFG-E8 (rMFG-E8) was administered to the injured artery using Pluronic gel to accentuate the effect on age-related vascular pathophysiology. The MFG-E8 level, leukocyte infiltration, and proinflammatory cell adhesion molecule (CAM) expression in the arterial wall were evaluated through immunohistochemistry. By using immunofluorescence and immunoblotting, the activation of the critical proinflammatory transcription factor nuclear factor (NF)-κB in the injured CCAs was analyzed. Immunofluorescence, immunoblotting, and quantitative real-time polymerase chain reaction were conducted using VSMCs isolated from the aortas of young and aged mice to assess NF-κB nuclear translocation, NF-κB-dependent gene expression, and cell proliferation. The extent of intimal–medial thickening in the injured vessels was analyzed morphometrically. Finally, Transwell migration assay was used to examine VSMC migration.

**Results:**

Endogenous MFG-E8 expression in aged CCAs was significantly induced by ligation injury. Aged CCAs treated with rMFG-E8 exhibited increased leukocyte extravasation, CAM expression, and considerably increased NF-κB activation induced by rMFG-E8 in the ligated vessels. Exposure of early passage VSMCs from aged aortas to rMFG-E8 substantially increased NF-κB activation, proinflammatory gene expression, and cell proliferation. However, rMFG-E8 attenuated VSMC migration.

**Conclusions:**

MFG-E8 promoted the proinflammatory phenotypic shift of aged VSMCs and arteries, rendering the vasculature prone to vascular diseases. MFG-E8 may constitute a novel therapeutic target for retarding the aging processes in such vessels.

**Electronic supplementary material:**

The online version of this article (10.1186/s12929-019-0559-0) contains supplementary material, which is available to authorized users.

## Background

Arterial aging is a major factor contributing to increases in cardiovascular disease–related morbidity and mortality [1–5]. Aged arteries are characterized by dilated lumen, diffuse intimal–medial thickening, and vascular stiffness in large elastic arteries [6, 7]. Accumulating evidence suggests that these age-associated structural and functional alterations in the cells and extracellular matrix (ECM) of the vascular wall are attributable to chronic low-grade arterial inflammation driven by the enhanced angiotensin II (Ang II) signaling cascades that accompany advancing age [3, 7–10]. By binding to its receptor, Ang II receptor type 1, Ang II initiates several intracellular signaling pathways that activate the inflammatory transcription factor nuclear factor (NF)-κB in aged endothelial cells and vascular smooth muscle cells (VSMCs), leading to the transcription of proinflammatory genes [11–14]. The microenvironment enriched with inflammatory profiles subsequently induces the phenotypic shift of VSMCs from the contractile to synthetic type, which is characterized by the attenuated expression of SMC-specific contractile proteins along with increased proliferation of VSMCs, migration from the media to the intima, and secretion of additional proinflammatory cytokines, chemokines, ECM proteins, and cell adhesion molecules (CAMs), such as monocyte chemotactic protein (MCP)-1, matrix metalloproteinase (MMP)-2 and − 9, collagen, intercellular adhesion molecule (ICAM)-1, and vascular CAM (VCAM)-1 [5, 15–18]. Increased CAM expression in the aged vasculature causes excessive leukocyte infiltration, triggering a positive inflammatory signaling loop [19, 20], rendering the aged vascular system prone to diseases. Therefore, targeting molecules involved in the Ang II–mediated signaling cascades in the vascular walls has therapeutic potential because it may attenuate age-associated chronic inflammation and retard aging processes in the vessels.

Milk fat globule–epidermal growth factor 8 (MFG-E8) is a secreted integrin-binding glycoprotein with multifunctional domains [21, 22]. In healthy and diseased vessels, MFG-E8 is expressed by endothelial cells, VSMCs, and macrophages [15, 23–25]. The anti-inflammatory function [26–29] and phagocytic role against apoptotic cells [25, 27] of MFG-E8 are well-documented. MFG-E8 is expressed in aged arteries and thus has been identified as a novel biomarker for arterial aging [5, 15]. MFG-E8 is induced by Ang II in VSMCs, and treatment of VSMCs with MFG-E8 increases MCP-1 expression [15]. Moreover, MFG-E8 can stimulate cultured VSMC proliferation and invasion through integrin/ERK1/2 signaling and MCP-1 secretion, respectively [5, 15]—both of which are hallmarks of age-associated vascular remodeling, which leads to intimal–medial thickening of vascular walls. In contrast to the anti-inflammatory function of MFG-E8 that is usually acknowledged, these results suggest a proinflammatory role of MFG-E8 in aging arteries. However, no evidence of the direct regulation of proinflammatory responses in aging vessels by MFG-E8 has been published.

In the present study, we established the molecular link connecting MFG-E8 with the proinflammatory profiles of aging arterial walls and VSMCs. To address the role of MFG-E8 in age-associated inflammatory remodeling, the common carotid arteries (CCAs) of aged mice were subjected to ligation injury to accelerate age-associated vascular remodeling. Recombinant MFG-E8 (rMFG-E8) was exogenously delivered to the injured arteries to further potentiate its effect on age-related vascular pathophysiology. The expression of endogenous MFG-E8 in aged CCAs was upregulated and thus higher than that in younger arteries; the ligation injury elevated MFG-E8 content in the aged vascular walls. Application of rMFG-E8 to the ligated CCAs of aged mice significantly increased leukocyte accumulation and ICAM-1 and VCAM-1 expression in the intima and media of the vessels. Moreover, the treatment of the ligated CCAs of aged mice and cultured VSMCs derived from aged aortas with rMFG-E8 increased NF-κB activation and the expression of proinflammatory genes downstream of NF-κB. Notably, exogenous MFG-E8 did not significantly increase intimal–medial thickening in the aged CCAs, a finding attributable to rMFG-E8 impairment of VSMC migration.

## Methods

### Animal surgery

Young (3 months old) and aged (18 months old) FVB/NJ mice were subjected to complete CCA ligation on the left side [30, 31]. The mice were first anesthetized with isoflurane inhalation. The left CCA was then exposed and excised from the surrounding connective tissue. Subsequently, a 6–0 silk suture was tightly ligated immediately proximal to the carotid bifurcation. In some animals, 50 μL of F127 Pluronic gel (BASF, Ludwigshafen, Germany), with or without 2 μg/mL mouse rMFG-E8 (R&D Systems, Minneapolis, MN, USA) was applied to the carotid artery periadventitially immediately after ligation. The endotoxin level in rMFG-E8 was evaluated using a Limulus Amebocyte Lysate assay kit (GenScript, Piscatway, NJ, USA) before the aforementioned application to ensure that the endotoxin levels was < 0.1 endotoxin unit (EU) per milligram (EU/mg) of protein. We have previously observed that Pluronic F127 gel does not noticeably influence vascular remodeling after CCA ligation [31, 32]. Each experimental group comprised 5–8 mice. All animal procedures were approved by the Chang Gung University Institutional Animal Care and Use Committee (IACUC Approval No: CGU15–098).

### Tissue collection and morphometric analysis

Mice were intracardially perfused with 10% formalin 4 and 21 days after the ligation injury. The CCAs were harvested and embedded in paraffin. For each animal, 5-μm-thick cross-sections within the first millimeter proximal to the ligature were stained with Verhoeff–van Gieson elastic stain. Subsequently, the intimal and medial areas were measured using Image-Pro Plus (Media Cybernetics, Rockville, MD, USA) as described in the literature [31, 32]. Human vessel specimens were obtained from aortic biopsies of young (20 years) and aged (60 years) patients with acute aortic dissection. All protocols involving human tissues were approved by the Institutional Review Board of Chang Gung Memorial Hospital (IRB No. 201701920A3).

### Immunohistochemistry and quantitative analysis

For each animal, three sections at 200-μm intervals within the first millimeter proximal to the ligature were selected for immunohistochemistry (IHC). The sections were incubated with anti-MFG-E8 (1:1000, R&D Systems), anti-CD45 (i.e., anti-leukocyte common antigen, 1:100, BD Pharmingen, San Jose, CA, USA), anti-ICAM-1 (1:200, BD Pharmingen), anti-VCAM-1 (1:10000, Santa Cruz Biotechnology, Dallas, TX, USA), and anti-Ki-67 (1:1000, Abcam, Cambridge, UK) antibodies. The human aortic sections were incubated with anti-ICAM-1 (1:300, Santa Cruz Biotechnology). The sections were subsequently incubated with appropriate biotinylated secondary antibodies and analyzed with an avidin–biotin immunoperoxidase system (Vector Laboratories, Burlingame, CA, USA). A liquid diaminobenzidine substrate chromogen system (Dako, Carpinteria, CA, USA) was used for detection. For quantitative analysis, digital images of the paraffinized sections were captured. To assess the levels of MFG-E8, ICAM-1, and VCAM-1, the mean optical density in the combined intimal and medial (intima+media) area in each section was obtained using Image-Pro Plus. For evaluating the proliferating cells, the percentage of Ki-67^+^ cells was defined as the proliferation index. For quantitative evaluation of leukocyte infiltration, the positive staining area for CD45 was identified using an automated programmed segmentation procedure in Image-Pro Plus. The intima+media region was manually traced. In the traced intima+media area, the percentage of the area that was positively stained was assessed. The data from 3 to 8 mice in each group were averaged. The average values ± standard errors of the mean (SEMs) are presented.

### VSMC culture and treatment

Mouse VSMCs were enzymatically isolated as described in the literature [33]. In brief, the thoracic aortas of the young and aged mice were isolated, and the adventitia and intima were removed from the vessels. The aortae were cut into 1–2-mm pieces and placed into tubes containing 2 mg/mL collagenase II (Worthington Biochemical, Lakewood, NJ, USA) at 37 °C for 3–5 h. The isolated cells were washed with and plated in complete Dulbecco’s Modified Eagle’s Medium (DMEM). To ensure the purity of the isolated cells, > 95% of the cells had to be positive for the two specific smooth muscle cell markers: smooth muscle α-actin and smooth muscle myosin heavy chain for them to be used [34]. Early passage VSMCs were treated with Ang II (1 μM, R&D Systems), tumor necrosis factor-α (TNF-α, 20 ng/mL, R&D Systems), or mouse MFG-E8 (250 ng/mL, R&D Systems) for 24 h prior to the subsequent experiments. Human aortic smooth muscle cells (hAoSMCs) were purchased from Lonza (Basel, Switzerland) and cultured in SmGM-2 medium (Lonza) for 24 h in either the presence or absence of human MFG-E8 (250 ng/mL, R&D systems).

### Immunofluorescence analysis and confocal microscopy

To locate MFG-E8 and phosphorylated NF-κB p65 in the mouse and human arteries, the paraffinized sections were deparaffinized, permeabilized with 0.1% Triton-X 100 in phosphate-buffered saline (PBS-T) for 10 min, blocked with 5% normal goat serum in PBS-T, and incubated with primary antibodies against MFG-E8 (1:200, R&D Systems) and NF-κB p65 phosphorylated at Ser536 (1:200, Cell Signaling Technology, Danvers, MA, USA) overnight at 4 °C followed by an additional incubation of Alexa Fluor 488– and 594–conjugated secondary antibodies (1:100, Thermo Fisher Invitrogen, Waltham, MA, USA) for 1 h at room temperature. VSMCs derived from young and aged aortas were washed in warm PBS and fixed in 1.75% paraformaldehyde for 15 min after the various treatments. After blocking, the cells were incubated with primary antibodies against MFG-E8 and NF-κB p65 phosphorylated at Ser536 followed by the appropriate secondary antibodies. Confocal images were captured using a Zeiss LSM 780 confocal scanning microscope (Oberkochen, Germany), and the images were processed in Photoshop CS6 (Adobe, San Jose, CA, USA).

### Western blotting

MFG-E8-treated VSMCs were homogenized in 1× Laemmli Sample Buffer (Sigma-Aldrich, St. Louis, MO, USA). The CCAs of the aged mice were homogenized using cold lysis buffer (50 mM Tris–HCl, pH 7.5; 150 mM NaCl, 1% NP-40, and 5 mM ethylenediaminetetraacetic acid) that was supplemented with a protease inhibitor cocktail (Sigma-Aldrich) [30]. Cell and tissue lysates were electrophoresed on sodium dodecyl sulfate polyacrylamide gel electrophoresis gels under reducing conditions and transferred to nitrocellulose membranes. These membranes were probed with antibodies either against NF-κB p65 phosphorylated at Ser536 (1:1000, Cell Signaling Technology), against NF-κB p65 (1:1000, Cell Signaling Technology), against ICAM-1 (1:1000, GeneTex, Irvine, CA, USA), against VCAM-1 (1:1000, GeneTex), or against proliferating cellular nuclear antigen (PCNA; 1:1000, Sigma-Aldrich) overnight, and then with horseradish peroxidase–conjugated secondary antibodies (1:12000; Bio-Rad, Hercules, CA, USA) for 1 h at room temperature. The blots were then developed by using enhanced chemiluminescence (Thermo Scientific, Rockford, IL, USA).

### Transwell migration assay

VSMCs isolated from the aortas of aged mice were grown in growth medium until they reached 70–80% confluence. The cells were then treated with rMFG-E8 overnight, followed by serum starvation for 24 h. The cells were subsequently trypsinized, and 3000 cells were seeded into the upper chamber of wells (pore size, 8 μm) in 24-well Transwell plates (Corning, Corning, NY, USA) and incubated with 800 μL of platelet-derived growth factor (PDGF)-BB (10 ng/mL) in DMEM in the lower chamber to induce VSMC migration. After 6 h of incubation, VSMCs that had migrated into the lower chambers were stained with calcein-AM and measured using the fluorescence intensity of the calcein-AM and a SpectraMax M5 microplate reader (Molecular Devices, Sunnyvale, CA, USA) set at 485/535 nm (excitation/emission).

### Quantitative real-time polymerase chain reaction

Total RNA from cells was isolated using TRIzol (Thermo Fisher Invitrogen). Total RNA from each sample was reverse transcribed with a First Strand cDNA Synthesis Kit (GE Healthcare Life Sciences, Marlborough, MA, USA) according to the manufacturer’s instructions. Quantitative real-time polymerase chain reaction (PCR) was performed using multiple sets of quantitative PCR primers (mouse ICAM-1 primers: forward, 5′-AGTGAGGAGGTGAATGTATAAG-3′, reverse, 5′-ATGTGGAGGAGCAGAGAA-3′; mouse VCAM-1 primers: forward, 5′-GGAGACTACACTGATGAAGA-3′, reverse, 5′-CGAGGCAAACAAGAGATTT-3′; mouse TNF-α primers: forward, 5′-CAACTACTCAGAAACACAAGAT-3′, reverse, 5′-GCAGAACTCAGGAATGGA-3′; mouse inducible NO synthase (iNOS) primers: forward, 5′-CAGAAGCAGAATGTGACC-3′, reverse, 5′-GTAGTAGTAGAATGGAGATAGGA-3′) and a SensiFAST SYBR No-ROX Kit (Bioline, London, UK) on a CFX96 Real-Time PCR Detection System (Bio-Rad). Quantitative real-time PCR was performed on the VSMCs that were derived from the young and aged mice and incubated with rMFG-E8. Each quantitative real-time PCR was performed at least three times, and the representative results are expressed as the change relative to the control using the standard 2^−ΔΔ*Ct*^ method [35], where *Ct* is the number of cycles required to reach the threshold for the target gene subtracted from the number of cycles required to reach the threshold for a control housekeeping gene (glyceraldehyde 3-phosphate dehydrogenase, Gapdh in this study).

### Statistical analysis

After tests for normality and equal variance had been conducted, appropriate statistical analyses were performed. The type of analysis used is indicated in the legend of each figure. For in vivo experiments, the data are presented as means ± SEM. For in vitro experiments, data are expressed as means ± standard. Student’s *t* test or a one-way analysis of variance followed by a post hoc test was conducted for data analysis in GraphPad Prism (GraphPad, San Diego, CA, USA). *P* > 0.05 was considered nonsignificant.

## Results

### Elevated MFG-E8 levels in aged carotid arteries increase leukocyte infiltration and CAM expression

**Fig. 1:**
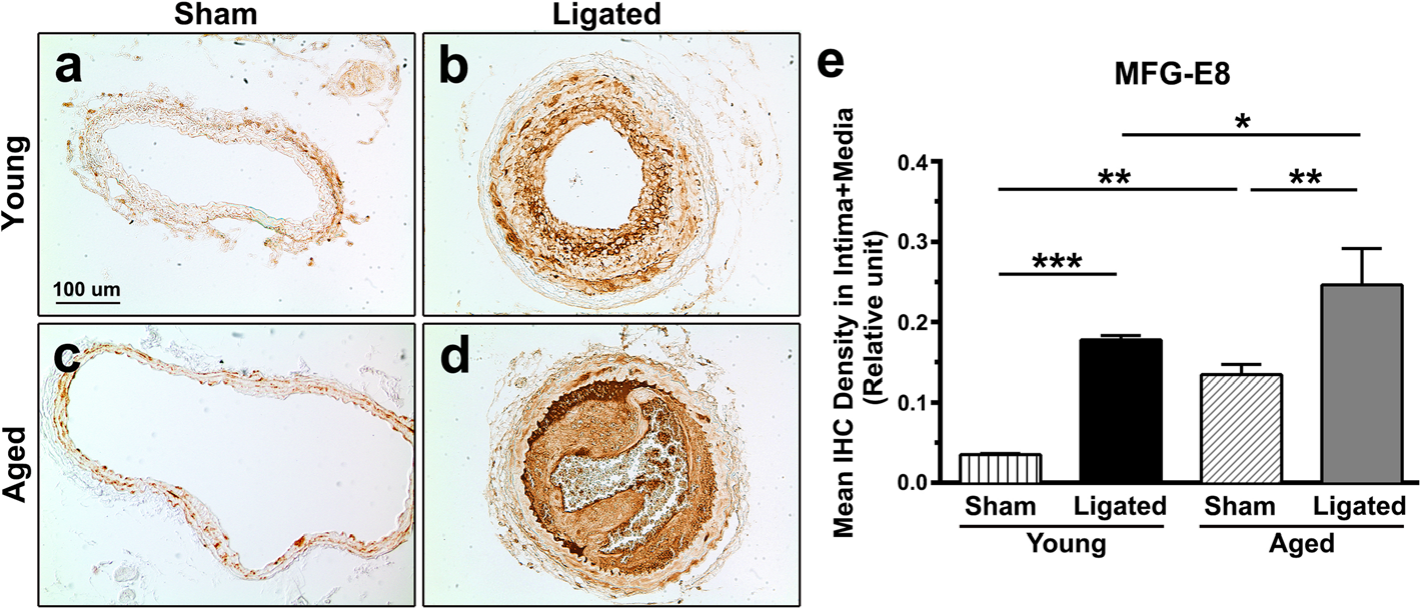
MFG-E8 is elevated in aged arteries. Sections of left CCA, 21 days after ligation, were stained with antibodies against MFG-E8. Representative IHC images of MFG-E8 in CCAs of young (**a**, **b**) and aged (**c**, **d**) mice 21 days after ligation; bar, 100 μm. **e** Quantification of immunostaining intensity of MFG-E8 in the intimal–medial area 21 days after ligation (*n* = 3 for each experimental group). Results are presented as mean ± SEM. * *P* < 0.05, **P < 0.01, and *** *P* < 0.001; one-way analysis of variance followed by Tukey’s multiple comparison test

Expression of MFG-E8 is elevated in aged aortic walls [5, 15]. To determine whether MFG-E8 levels are higher in aged CCAs, we examined MFG-E8 expression in carotid arteries in young and aged mice through IHC (Fig. 1). MFG-E8 expression was low in the sham-operated arteries of young mice (Fig. 1a) but much higher in those of aged mice (Fig. 1c and e). Subsequently, we ligated the CCAs to induce neointimal hyperplasia of the vessels by completely interrupting the blood flow [30, 31] and investigated whether a vascular lesion on an aged vessel further increases MFG-E8 levels. As illustrated in Fig. 1, in contrast to the sham-operated arteries of both young and aged mice, the ligation injury significantly increased MFG-E8 expression in the neointima and media of CCAs 21 days after ligation (Fig. 1b, d, and e). Notably, endogenous MFG-E8 levels in the ligated CCAs of aged mice were higher than in those of young mice. Therefore, to identify the role of MFG-E8 in arterial aging, exogenous rMFG-E8 was periadventitially delivered into the ligated aged CCAs via Pluronic gel to further elevate MFG-E8 content in the aged vessels. We previously demonstrated that a recombinant peptide could be delivered periadventitially via Pluronic gel into the tunica intima and media of a mouse carotid artery after surgery [32].

**Fig. 2:**
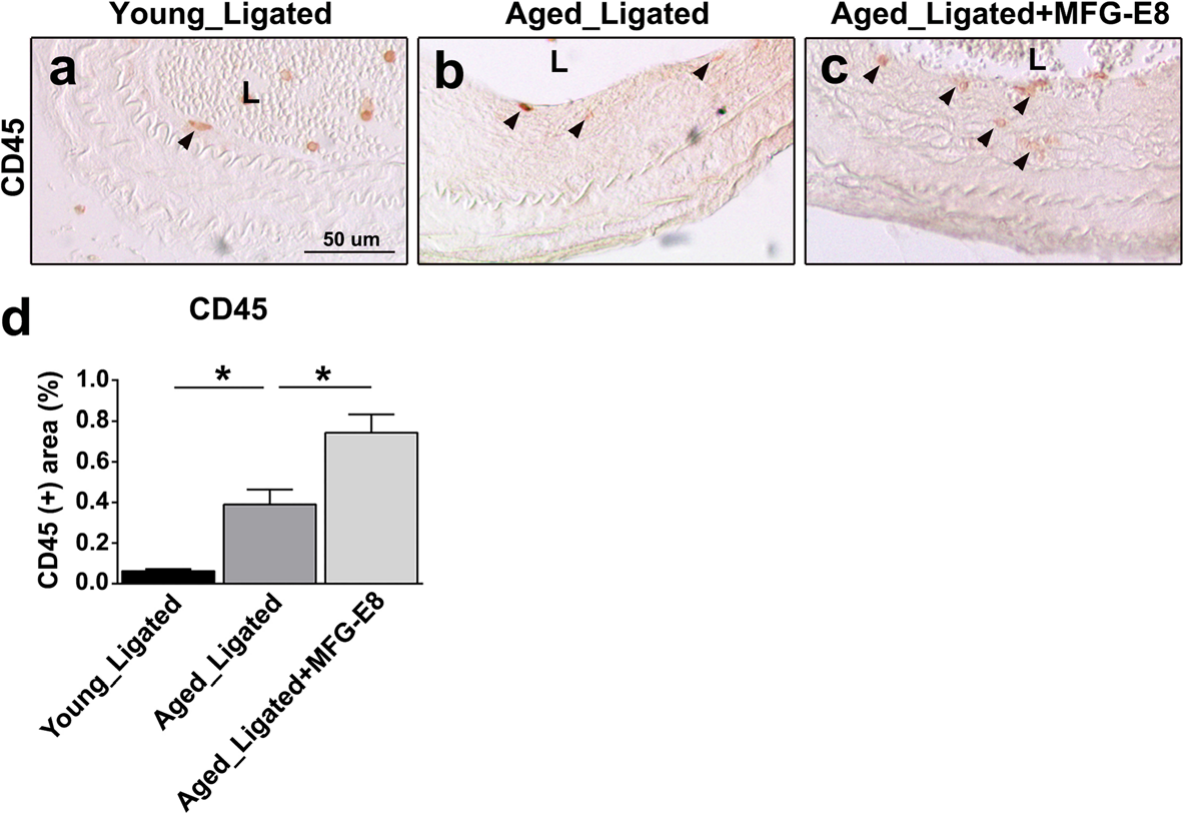
Exogenous treatment with MFG-E8 exacerbates leukocyte infiltration into ligated CCAs of aged mice. Sections of left CCA, 21 days after ligation, were stained with antibodies against the common leukocyte marker CD45. Representative IHC images of CD45 in the intima and media of young ligated CCAs (**a**), aged ligated CCAs (**b**), and aged ligated CCAs treated with rMFG-E8 (2 μg/mL) (**c**). Arrowheads indicate CD45^+^ cells. Lumens (L) of the ligated vessels are displayed; bar, 50 μm. **d** The percentages of CD45^+^ area in the intima and media were analyzed (young ligated: *n* = 3; aged ligated: *n* = 8; aged ligated + MFG-E8: *n* = 8). Data are presented in terms of the mean ± SEM. *P < 0.05; one-way analysis of variance followed by Tukey’s multiple comparison test

**Fig. 3:**
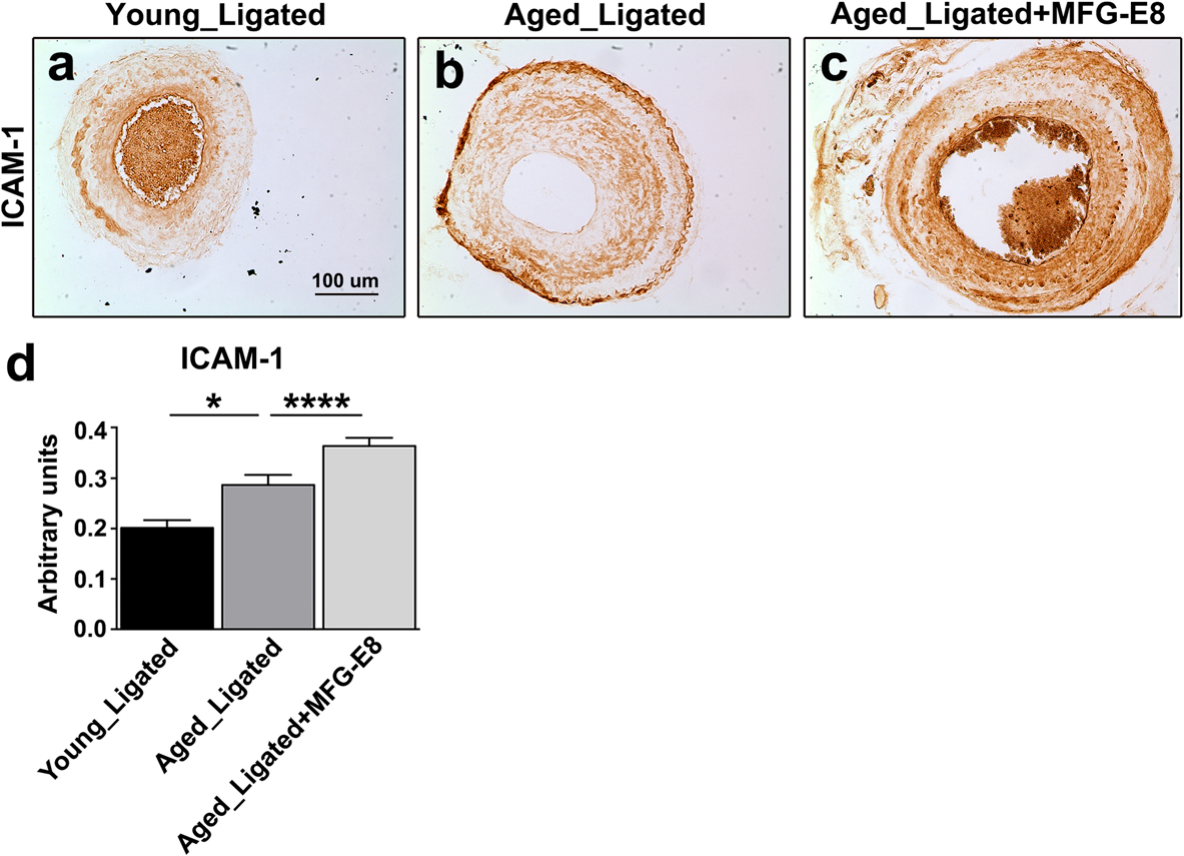
Treatment of aged CCAs with MFG-E8 increases ICAM-1 expression in the vascular wall. **a**–**c** Photographs depicting representative immunostaining of ICAM-1 in the left carotid artery from young (**a**) and aged (**b**) mice subjected to ligation injury 21 days after surgery. (**c**) rMFG-E8 (2 μg/mL) was delivered using Pluronic gel into the CCAs of aged mice immediately after complete ligation of the vessels; bar, 100 μm. **d** Quantitative analysis of immunostaining intensities of ICAM-1 in the intimal–medial area 21 days after ligation (young ligated: *n* = 3; aged ligated: *n* = 6; aged ligated + MFG-E8: *n* = 4). Data are presented as mean ± SEM. *P < 0.05 and **** *P* < 0.0001; one-way was analysis of variance followed by Tukey’s multiple comparison test

**Fig. 4:**
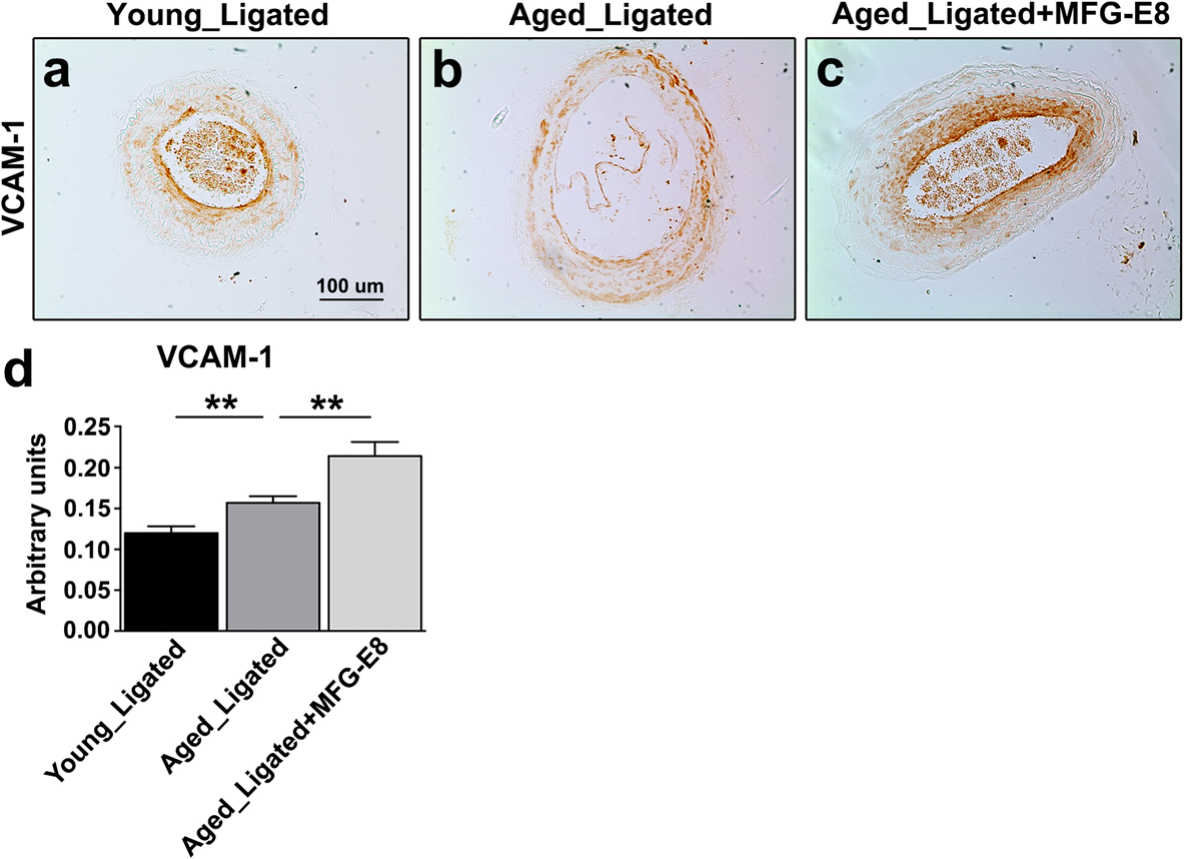
Exogenous MFG-E8 application to aged CCAs upregulates VCAM-1 expression. **a**–**c** Sections of left CCA, 21 days after ligation, were immunostained with antibodies against VCAM-1. Representative photomicrographs of ligated CCA sections from young (**a**) and aged (**b**) mice at 21 days after ligation. (**c**) rMFG-E8 (2 μg/mL) was exogenously applied to the ligated carotid arteries of aged mice; bar, 100 μm. **d** Quantitative analysis of the immunostaining intensities of VCAM-1 in the intimal–medial area 21 days after ligation (young ligated: *n* = 3; aged ligated: *n* = 6; aged ligated + MFG-E8: *n* = 4). Data are presented in terms of the mean ± SEM. **P < 0.01; one-way analysis of variance followed by Tukey’s multiple comparison test

We next investigated whether exogenous application of rMFG-E8 to the injured CCAs of aged mice exacerbates leukocyte extravasation and increases ICAM-1 and VCAM-1 expression. In a previous study, increased leukocyte adhesion and influx were associated with increased age-related chronic inflammation [36]; this inflammation is a crucial initial step in age-associated vascular remodeling. Immunostainings indicated that CD45^+^ leukocyte infiltration in the ligated arteries of aged mice was significantly increased (Fig. 2a, b, and d), in contrast to that in those of young mice. Moreover, IHC revealed that the additional MFG-E8, the exogenous rMFG-E8, substantially increased the number of CD45^+^ leukocytes in the intima and media of aged CCAs (Fig. 2c and d). The increase in leukocyte influx is attributable to upregulated VCAM-1 and ICAM-1 expression [37]. Therefore, we assessed the ICAM-1 and VCAM-1 content in the CCAs of the young and aged mice. Compared with those of young mice, the ICAM-1 (Fig. 3) and VCAM-1 (Fig. 4) levels in the ligated arteries of aged mice were significantly higher. Treatment of the ligated CCAs of aged mice with rMFG-E8 considerably increased the levels of ICAM-1 (Fig. 3c and d) and VCAM-1 (Fig. 4c and d) in the vascular wall. Together, our in vivo results demonstrate that MFG-E8 promotes the proinflammatory state in the vascular pathology of aged arteries by increasing leukocyte infiltration and CAM expression.

### MFG-E8 exacerbates age-related NF-κB activation and NF-κB-dependent proinflammatory gene expression in aged CCAs and VSMCs

**Fig. 5:**
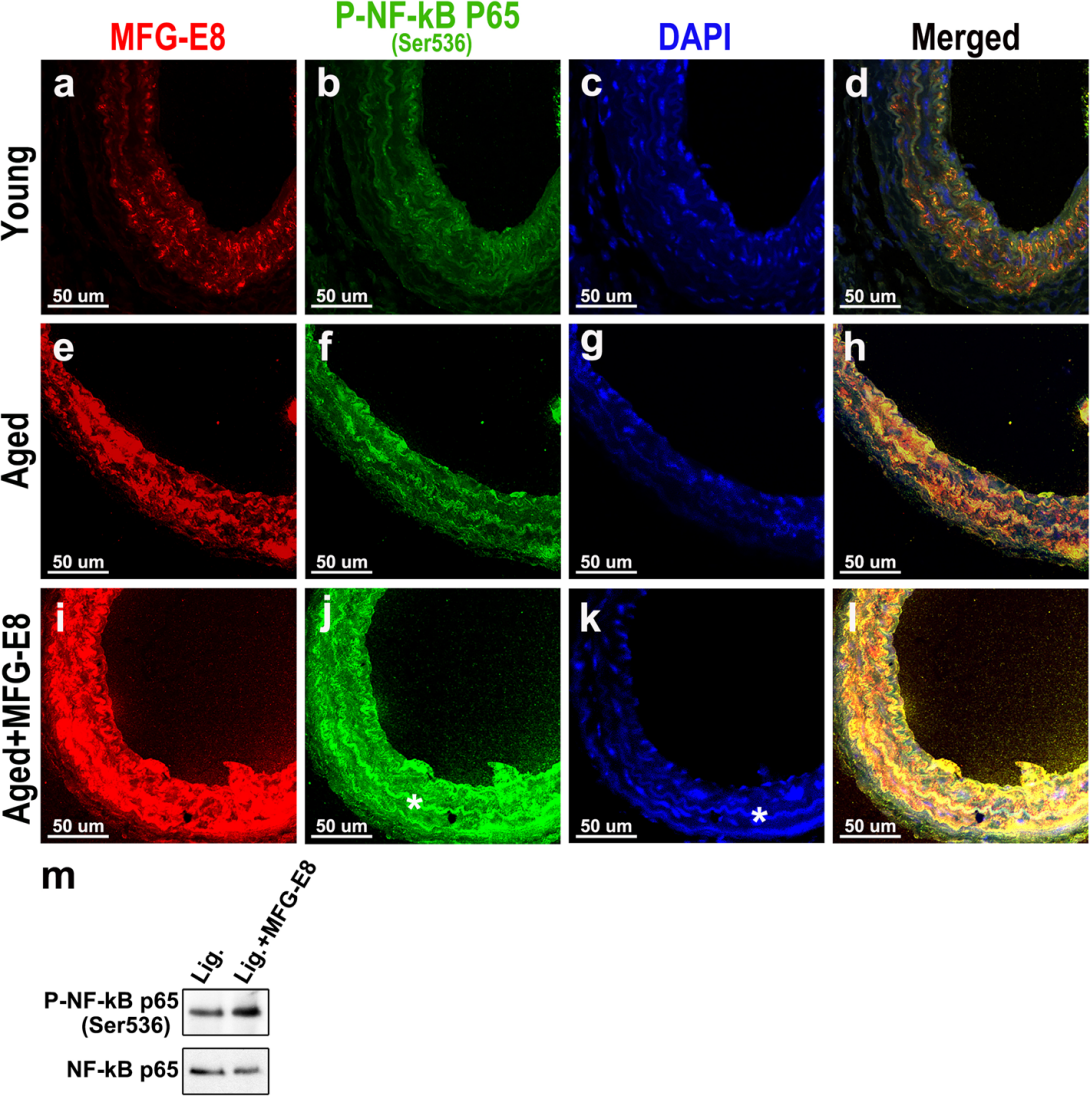
Exogenous MFG-E8 increases NF-κB activation in injured aged arteries. **a**–**l** CCAs of young and aged mice were ligated, with or without rMFG-E8 (2 μg/mL) treatment for 4 days. Representative dual immunofluorescence photographs of MFG-E8 (**a**, **e**, and **i**) and phosphorylated NF-κB p65 (Ser536) (**b**, **f**, **j**) in ligated CCA sections. The merged images depict an association (yellow) of MFG-E8 (red) and phosphorylated NF-κB p65 (Ser536) (green) (**d**, **h**, and **l**); bar, 50 μm. **m** CCAs of aged mice were ligated, with or without rMFG-E8 treatment for 4 days. Proteins were extracted from 10 pooled CCAs per group for Western blotting and examined using antibodies against NF-κB p65 phosphorylated at Ser536 and NF-κB p65

ICAM-1 and VCAM-1 are the target genes of the inflammatory transcription factor NF-κB [38, 39]. Our data revealed that ICAM-1 and VCAM-1 levels in the aged arterial wall were elevated in response to exogenous rMFG-E8. This finding suggests that MFG-E8 promotes NF-κB activation during the pathological vascular remodeling that occurs during aging. Therefore, NF-κB p65 phosphorylated at Ser536, required for the nuclear translocation of NF-κB and downstream signaling initiation [40], was evaluated through immunofluorescence and Western blotting to compare the NF-κB activity in the ligated CCAs of mice treated with and without rMFG-E8. Dual immunofluorescence demonstrated that both phosphorylated NF-κB p65 and MFG-E8 expression were markedly higher and closely associated in the ligated arteries of aged mice compared with those of young mice (Fig. 5a, b, d, e, f, and h). Subsequently, we investigated whether MFG-E8 directly initiates the transactivation of NF-κB in aged vessels. Figure 5j and l reveal that the delivery of MFG-E8 into the ligated vessels of aged mice substantially increased the level of phosphorylated NF-κB p65 and its co-expression with MFG-E8. The effects of MFG-E8 on the phosphorylation of NF-κB p65 in aged CCAs were confirmed through immunoblotting (Fig. 5m). The age-associated increase in MFG-E8 also occurs in the human aorta [15]. To confirm the association between MFG-E8 and inflammation in aged human arteries, we evaluated the expression of MFG-E8 and phosphorylated NF-κB p65 in human vessels using immunofluorescence. Compared with those of young patients, the aortas of older patients exhibited higher staining and co-expression of MFG-E8 and phosphorylated NF-κB p65 (Additional file 1: Figure S1a, b, d, f, g, and i). Additionally, similar to the arteries of aged mice, the expression of ICAM-1 (the downstream molecule of NF-κB) increased in the aged human aortas (Additional file 1: Figure S1e and j).

**Fig. 6:**
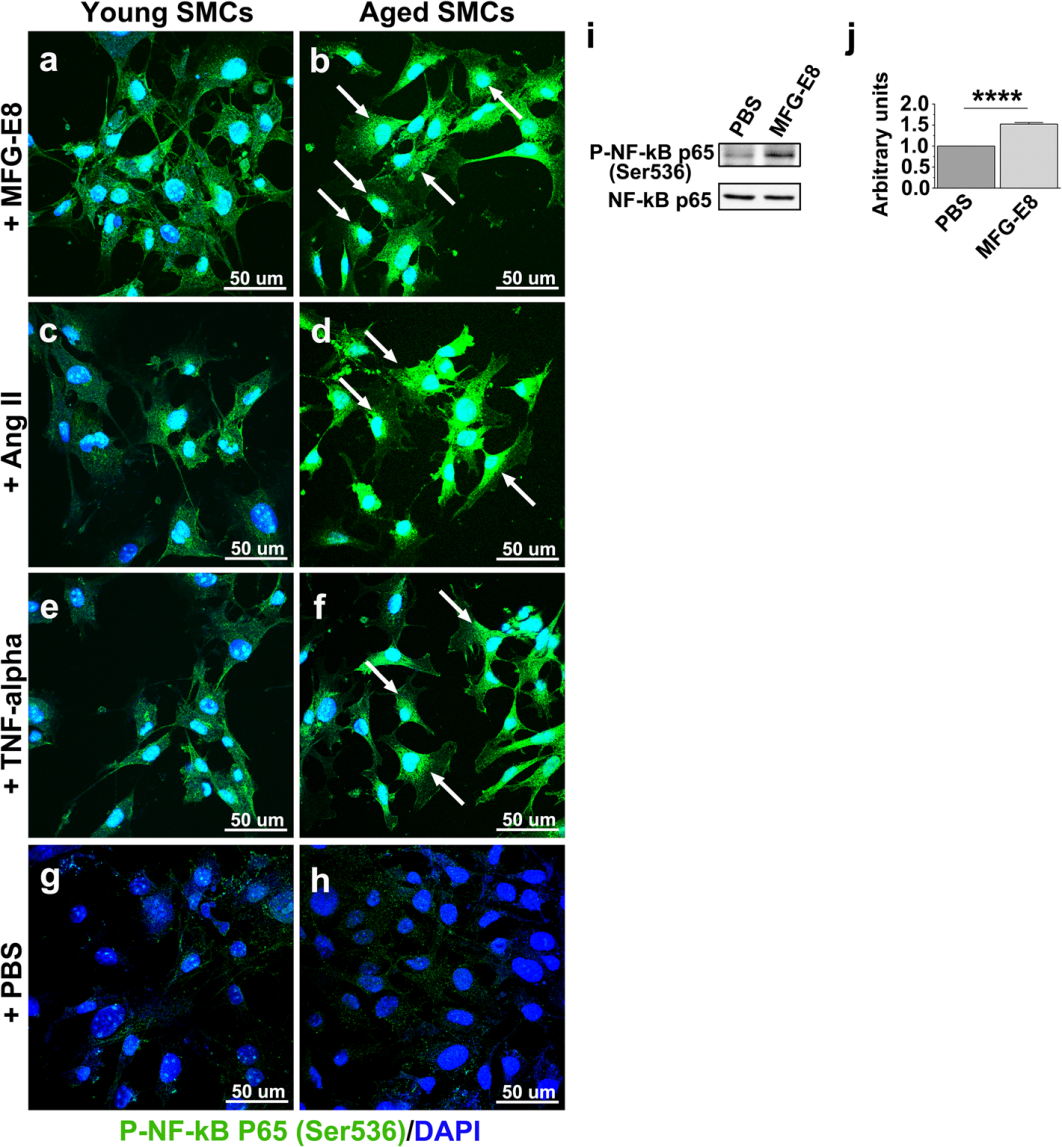
MFG-E8 increases phosphorylation and nuclear translocation of NF-κB p65 in aged VSMCs. **a**–**h** After being incubated with rMFG-E8 (**a**–**b**), Ang II (1 μM) (**c**–**d**), TNF-α (20 ng/mL) (**e**–**f**), and PBS (**g**–**h**) for 24 h, VSMCs isolated from young and aged aortas were immunostained with antibody against phosphorylated NF-κB p65 (Ser536). Arrows indicate nuclear translocation of NF-κB p65 in cells. Three independent experiments were performed; each experiment was repeated with similar results. **i**–**j** VSMCs isolated from the aortas of aged mice were cultured, either with or without rMFG-E8 (250 ng/mL), for 24 h. (**i**) Cell lysates were analyzed with immunoblotting with antibodies specific for NF-κB p65 phosphorylated at Ser536 and NF-κB p65. (**j**) Levels of phosphorylated p65, normalized to that of total p65 (*n* = 4), were analyzed. Data are presented as mean ± standard deviation. ****P < 0.0001; Student’s *t* test

VSMCs migrating from the media are the predominant cell type in the intima of the aged vessels [41, 42], and aged VSMCs express more VCAM-1 and MCP-1—the downstream molecules of MFG-E8—than do young VSMCs [43]. Therefore, we investigated the role of MFG-E8 in NF-κB activation in VSMCs. We used early passage primary VSMCs derived from the aortas of young and aged mice to detect an association between MFG-E8 and NF-κB activation. As depicted in Fig. 6, treatment of aged VSMCs with Ang II and TNF-α induced higher fluorescence intensity of phosphorylated NF-κB p65 and more nuclear translocation of p65 than it did in young VSMCs. Notably, administration of MFG-E8 alone to aged VSMCs initiated a significantly more nuclear translocation of phosphorylated NF-κB p65 than it did to young cells (Fig. 6a and b). Immunoblotting analyses indicated that the treatment of aged VSMCs with 250 ng/mL rMFG-E8 for 24 h resulted in a significant increase in NF-κB activation, as evidenced by increased NF-κB p65 phosphorylated at Ser536 (Fig. 6i and j). Similar results were noted for hAoSMCs treated with Ang II to mimic aging [15] (Additional file 1: Figure S1k). Together, these results demonstrate that MFG-E8 is as efficient as Ang II and TNF-α in initiating NF-κB signaling in VSMCs.

**Fig. 7:**
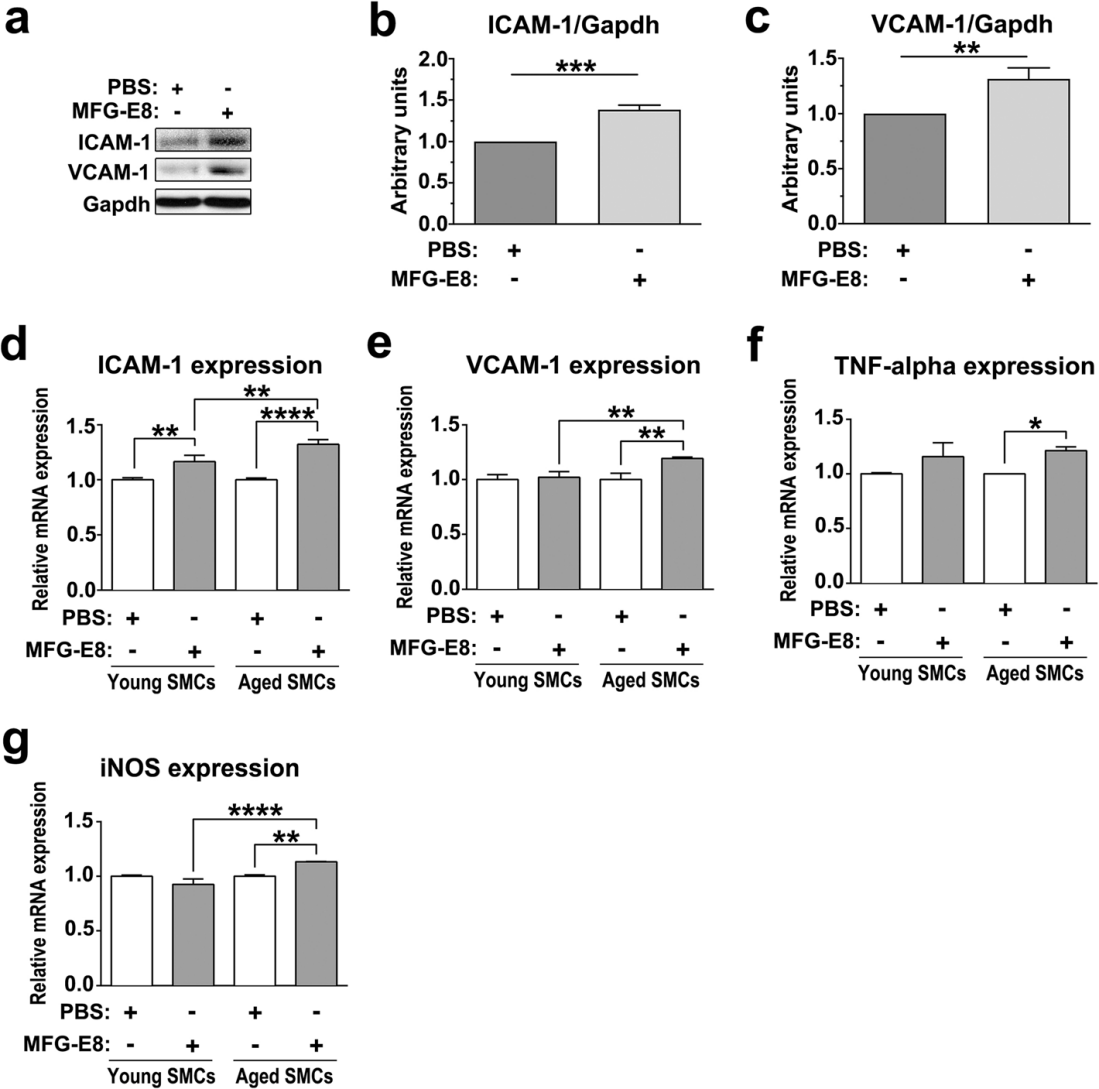
MFG-E8 increases transcription of NF-κB-dependent proinflammatory genes in aged VSMCs. **a**–**c** VSMCs isolated from the aortas of aged mice were cultured with or without rMFG-E8 (250 ng/mL) for 24 h. **a** The protein expression of ICAM-1 and VCAM-1 in aged VSMCs was evaluated through immunoblotting. The quantitative analysis results for ICAM-1 (**b**) and VCAM-1(**c**) are normalized to Gapdh (*n* = 3). **d**–**g** VSMCs derived from young and aged mice were treated with 1% fetal bovine serum in either the presence or absence of rMFG-E8 for 16 h after starvation. The transcript expression of downstream genes of NF-κB including ICAM-1 (**d**), VCAM-1 (**e**), TNF-α (**f**), and iNOS (**g**) in VSMCs treated with rMFG-E8 was evaluated through quantitative real-time PCR (*n* = 3). Data are presented as mean ± standard deviation. *P < 0.05, **P < 0.01, ***P < 0.001, and ****P < 0.0001, one-way analysis of variance followed by Tukey’s multiple comparison test

We next investigated a panel of genes to determine whether MFG-E8 also regulates NF-κB-dependent inflammatory genes in VSMCs. Consistent with the in vivo results (Figs. 3 and 4), MFG-E8 increased the protein expression of ICAM-1 (Fig. 7a and b) and VCAM-1 (Fig. 7a and c) in aged VSMCs. MFG-E8 also induced expression of VCAM-1 in Ang II-treated hAoSMCs (Additional file 1: Figure S1 l). In addition to ICAM-1 and VCAM-1, we evaluated several NF-κB-dependent proinflammatory genes previously shown through quantitative real-time PCR to be upregulated during arterial aging [44]. MFG-E8 exposure induced a significant upregulation in the mRNA levels of ICAM-1 (Fig. 7d) but not of VCAM-1 (Fig. 7e), TNF-α (Fig. 7f), or iNOS (Fig. 7g) in the VSMCs derived from young mice. By contrast, MFG-E8 markedly increased ICAM-1, VCAM-1, TNF-α, and iNOS mRNA expression in the VSMCs isolated from aged mice (Fig. 7d–g). Taken together, these results demonstrate that aged VSMCs are much more susceptible than young VSMCs to MFG-E8 challenge; therefore, VSMCs toward the proinflammatory phenotype in the pathological process of arterial aging.

### Exogenous MFG-E8 does not increase the ligation-induced intimal–medial thickening by impairing VSMC migration

Our data indicate that MFG-E8 promotes ICAM-1 and VCAM-1 expression and leukocyte infiltration into aged CCA intima and media and accelerates NF-κB activation and proinflammatory gene expression in aged VSMCs. These properties of MFG-E8 may lead to increased intimal–medial thickening of the ligated vascular wall after exogenous MFG-E8 treatment. We therefore subsequently evaluated through morphometric analysis the extent of vascular remodeling in the ligated CCAs of aged mice in the presence or absence of MFG-E8.

**Fig. 8:**
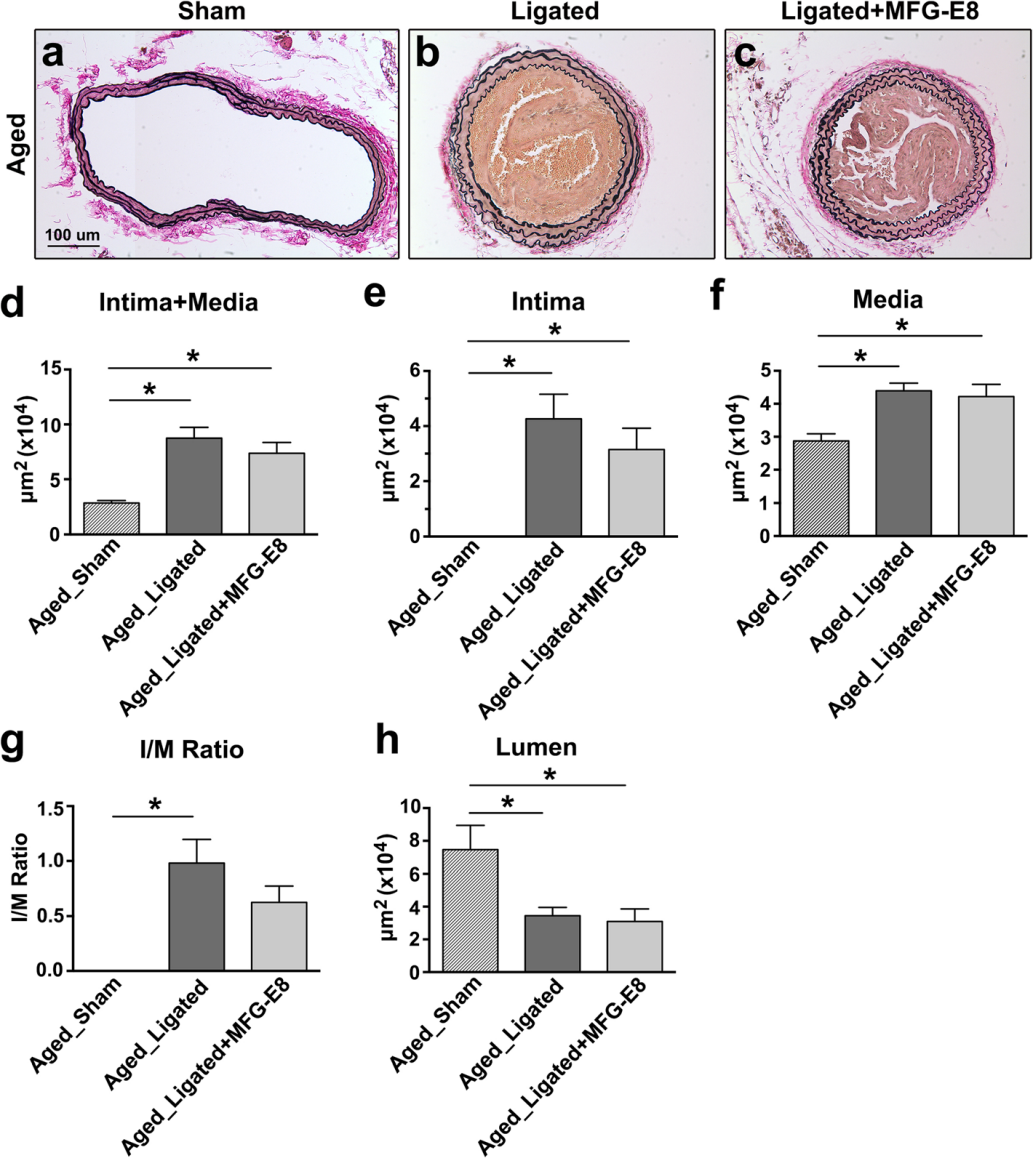
Exogenous MFG-E8 into carotid arteries dose not significantly increase ligation-induced vascular remodeling in aged mice. **a**–**c** Representative images displaying the cross-sectional areas of sham-operated (**a**) and ligated (**b**) carotid arteries of aged mice 21 days after ligation or sham operation. rMFG-E8 (2 μg/mL) was delivered using Pluronic gel into the CCAs of aged mice immediately after complete ligation of the vessels (**c**). Verhoeff–van Gieson staining was performed on all sections; bar, 100 μm. **d**–**h** Morphometric analyses of the areas of intima+media (**d**), intima (**e**), media (**f**), and lumen (**h**) and the I/M ratio (**g**) 21 days after ligation (*n* = 5–8 for each experimental group). Data are presented as mean ± SEM. *P < 0.05; one-way was analysis of variance followed by Tukey’s multiple comparison test

**Fig. 9:**
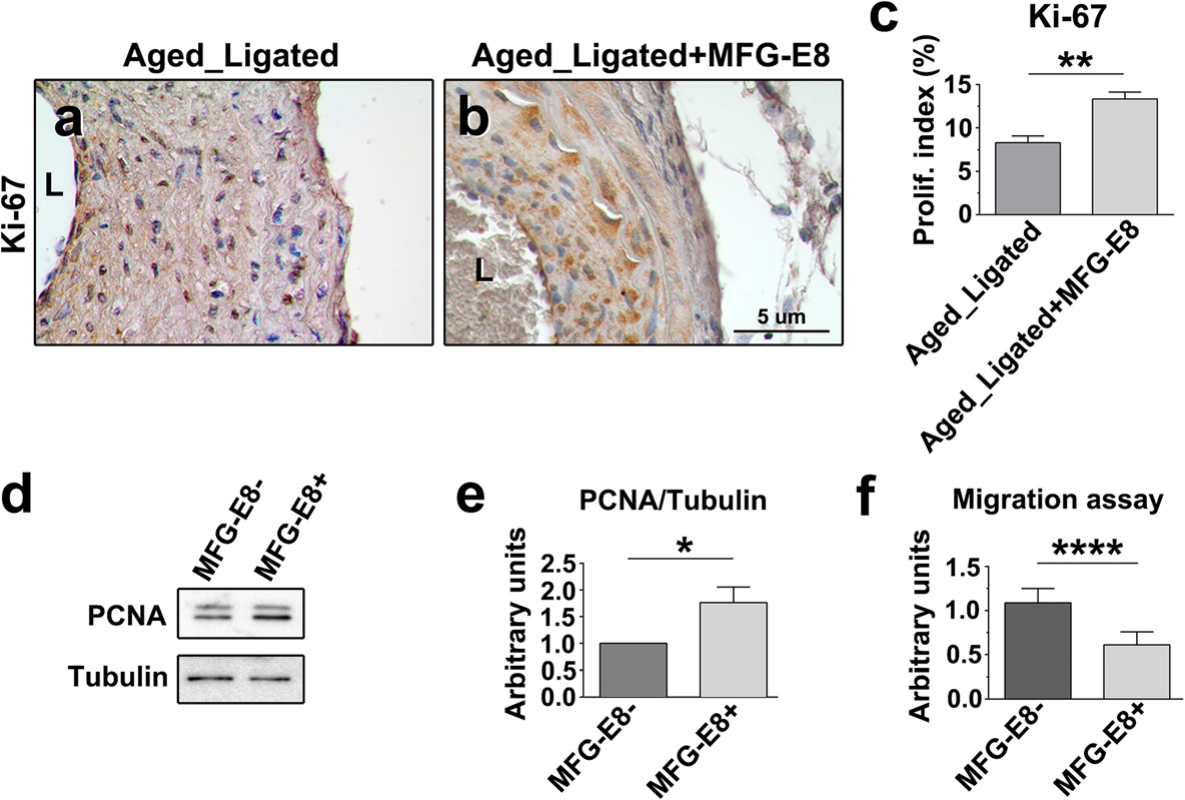
MFG-E8 increases VSMC proliferation but impairs VSMC migration. **a**–**b** Photomicrographs of the immunostaining of Ki-67 in cross-sections of ligated aged CCAs either with or without periadventitial delivery of rMFG-E8 (2 μg/mL) at 21 days after ligation; bar, 5 μm. **c** Quantitative IHC analysis of the proliferation index (Ki-67^+^ cells over total cell number) in the intimal–medial area of the vessel 21 days after ligation (*n* = 4 for each experimental group). Data are presented as mean ± SEM. **P < 0.01; Student’s *t* test. **d**–**e** VSMCs isolated from the aortas of aged mice were cultured either with or without MFG-E8 (250 ng/mL) for 24 h. Cell lysates were analyzed through immunoblotting with antibodies specific for PCNA. The PCNA level, normalized to that of tubulin, was quantified (*n* = 3). Data are presented as mean ± standard deviation. *P < 0.05; Student’s *t* test. **f** VSMCs derived from aged aortas were incubated either with or without MFG-E8 for 24 h. The cells were trypsinized and then seeded into the upper wells of 24-well Transwell plates with 8-μm pores for 6 h of incubation with PDGF-BB (10 ng/mL) in the lower chamber. The cells that had migrated into the lower chambers were stained with calcein-AM and quantified using the fluorescence intensity. Data are presented as mean ± standard deviation. ****P < 0.0001; Student’s *t* test

By the 21st day after ligation, the area of the intima and media and the intima-to-media (I/M) ratio were significantly higher and the lumen area notably lower in the ligated vessels compared with the sham-operated vessels (Fig. 8). Of note, no robust increase in intimal or medial area was observed in the carotid arteries treated with rMFG-E8 when compared with those not treated with rMFG-E8. By contrast, the intimal area and I/M ratio were slightly decreased (Fig. 8). Because VSMC proliferation and migration contribute to intima–media thickening after arterial injury [45, 46], we conducted IHC for Ki-67 to evaluate the level of VSMC proliferation in the ligated CCAs in the presence or absence of rMFG-E8. The percentage of Ki-67^+^ cells in the intimal–medial area was significantly higher after the application of rMFG-E8 (Fig. 9a–c). The level of PCNA expression in VSMCs isolated from the aorta of aged mice was then evaluated through immunoblotting. Western blotting revealed that treatment of VSMCs with rMFG-E8 for 24 h significantly increased PCNA levels in the cells (Fig. 9d and e), indicating that MFG-E8 promotes the proliferation of aged VSMCs and corroborating the result of a previous study [5]. We next investigated whether MFG-E8 affects VSMC migration using a Transwell assay. The migration in response to PDGF-BB was significantly lower in the primary VSMCs treated with rMFG-E8 than in those treated with the vehicle (Fig. 9f). Our data suggest that MFG-E8 promoted the proliferation of VSMCs but impaired their migration during ligation-induced vascular remodeling in aged arterial walls treated with additional MFG-E8.

## Discussion

We demonstrated that MFG-E8, a biomarker of arterial aging, can exacerbate proinflammatory responses in the aged arterial wall during pathological vascular remodeling. In vivo, endogenous MFG-E8 expression in aged carotid arteries was higher than that in young arteries, and ligation injury, particularly in aged mice, further upregulated MFG-E8 levels in injured CCAs (Fig. 1). Thus, endogenous MFG-E8 is potentially associated with the pathogenesis of aging arteries. To emphasize the role of MFG-E8 in age-related vascular remodeling, we administered rMFG-E8 to the injured arteries and observed that this protein promoted leukocyte influx (Fig. 2) and ICAM-1 (Fig. 3) and VCAM-1 (Fig. 4) expression in the arterial wall. Furthermore, exogenous MFG-E8 increased NF-κB activation in aged arteries and VSMCs (Figs. 5 and 6), increasing expression of NF-κB-dependent proinflammatory genes (Fig. 7). In summary, age-related pathological changes in the vessels were partly attributable to the proinflammatory phenotypic shift of VSMCs caused by MFG-E8.

Our results demonstrate that the treatment of aged carotid arteries and VSMCs with rMFG-E8 increases NF-κB activation. Activated NF-κB triggers the transcription of numerous proinflammatory genes—including cytokine, chemokine, reactive oxygen species, tissue-degrading enzyme, and adhesion molecule genes [47, 48]—initiating a forward feedback loop that sustains proinflammatory responses in the aged arterial wall, rendering arteries susceptible to vascular insults. In addition to Ang II, the transcriptional activity of NF-κB can be triggered by numerous stimuli, such as those elicited by oxidative stress, DNA damage, cytokines, and growth factors in the aging arterial wall [48, 49]. Furthermore, integrin αvβ3, the specific integrin receptor for MFG-E8, can mediate integrin-induced NF-κB activation in carotid arteries subjected to flow-induced vascular injury [50]. Inhibition of αvβ3 significantly suppresses macrophage infiltration and VCAM-1 expression in ligated arteries [50]. Therefore, we postulate that MFG-E8 acts as a trigger of NF-κB activation by ligating to αvβ3 integrin in aged vessels and VSMCs.

MFG-E8 has long been recognized as an anti-inflammatory mediator. MFG-E8 secreted by bone marrow–derived dendritic cells plays a protective role by aiding in the engulfment of apoptotic cells by the dendritic cells, attenuating proinflammatory responses during sepsis [51, 52]. Moreover, MFG-E8 can directly inhibit LPS-induced NF-κB activation in peritoneal macrophages [26, 28]. In the vasculature, MFG-E8 deficiency in macrophages causes defective clearance of apoptotic cells followed by increased production of proinflammatory cytokines, such as TNF-α and interleukin (IL)-12 as well as induces the synthesis of anti-inflammatory modulators, namely transforming growth factor β and IL-10, in the atherosclerotic vascular walls [25]. By contrast, studies have suggested that MFG-E8 exerts its proinflammatory effects in aged arteries through accumulation of its cleavage fragment, medin, an amyloidogenic peptide [53]. In the present study, we demonstrated that treatment of primary VSMCs with full-length MFG-E8 can initiate proinflammatory responses, indicating that in addition to its cleavage product, medin, full-length MFG-E8 itself can induce proinflammatory signaling in aged arteries and VSMCs. Additionally, we have demonstrated that MFG-E8 plays a proinflammatory role in aged arteries and VSMCs, but not in young vasculature or VSMCs, by directly promoting NF-κB activation. This indicates that age-related changes in VSMCs sensitize the cells to the proinflammatory effects of MFG-E8.

With regard to the roles of MFG-E8 in vascular inflammation, the differences between previous studies and the present investigation may be attributable to the disparate pathophysiological situations considered. Chronic cell apoptosis, necrosis, and subsequent inflammation are more common in atherosclerosis [54]. By contrast, cell apoptosis peaks only several hours after flow-cessation surgery and is followed by the swift phagocytosis of dead cells [55]. Furthermore, aged arterial walls with low-grade inflammation contain few apoptotic cells [3, 7–10, 56]. Taken together, in age-related vascular remodeling, MFG-E8 may exert primary effects by regulating VSMC behaviors rather than by mediating apoptotic cell clearance.

MFG-E8 increases the invasion capacity of aged VSMCs in a MCP-1-dependent manner [15]. VSMC migration from the media to intima is a complicated process comprising several mechanisms. MCP-1, the downstream molecule of MFG-E8, is a potent chemoattractant that induces VSMC migration [57]. MCP-1 levels increase in the intima with age [42], thus blocking the interaction between MCP-1 and its receptor markedly eliminates VSMC invasion in vitro [15]. Numerous studies have reported that MMP-2 and MMP-9 expression and activity are elevated in aged arterial walls [42, 58]. Such elevation potentially facilitates VSMC migration from the media to intima by degrading the subendothelial matrix in arterial walls [59, 60]. In addition to stimulating MMP-2 and MMP-9 activity, MFG-E8 may mediate VSMC migration through other mechanisms, such as cytoskeletal rearrangement [61], alterations in cell-to-matrix adhesiveness, motor protein activation, and changes in responses to chemoattractants [61]. In this study, exogenous application of rMFG-E8 to aged ligated CCAs and VSMCs increased VSMC proliferation but inhibited PDGF-BB-induced cell migration. We speculate that using VSMCs from different animal species (rat vs. mouse) led our results to contrast those of previous studies. We may need to evaluate, in the injured vessels of our animal model and in cultured mouse VSMCs, the expression and activity of MMP-2 and -9, remodeling of cytoskeletons, and other aforementioned factors to elucidate their effects on MFG-E8-mediated VSMC migration.

## Conclusions

We established a molecular link between MFG-E8 and NF-κB-dependent aging-related proinflammatory signaling in the arterial wall. By inducing neointimal hyperplasia in aged arteries and applying rMFG-E8 treatment, we confirmed that the age-associated increase in MFG-E8 levels in the arterial wall exacerbates the inflammatory responses in the vessels and promotes a proinflammatory phenotypic shift of VSMCs, rendering aged arteries prone to cardiovascular diseases. Therefore, the pharmacological inhibition of MFG-E8 may represent a novel therapeutic strategy for retarding the aging processes in such vessels.

## Additional file

Additional file 1: **Figure S1.** MFG-E8 abundantly expresses in aged human aortas and augments transactivation of NF-κB p65 in human VSMCs. (a–j) Representative IHC images display increased coexpression of MFG-E8 and phosphorylated NF-κB p65 (Ser536) as well as elevated ICAM-1 intensity in aged aortas. Arrows indicate the autofluorescence of the elastic laminae in the vessels; bar, 100 μm. (k–l) After treatment with rMFG-E8 (250 ng/mL) for 24 h, hAoSMCs were stimulated with Ang II (1 μM) for 24 h to mimic aging. (k) Cell lysates were analyzed through immunoblotting with antibodies specific for NF-κB p65 phosphorylated at Ser536 and NF-κB p65. Levels of phosphorylated p65, normalized to that of total p65 (*n* = 3), were analyzed. (l) The protein expression of VCAM-1 in hAoSMCs was evaluated through immunoblotting; the quantitative analysis results for VCAM-1, normalized to that of Gapdh, are displayed (*n* = 3). Data are presented as mean ± standard deviation. *P < 0.05 and **P < 0.01, one-way analysis of variance followed by Tukey’s multiple comparison test. (PDF 474 kb)

## Data Availability

Not applicable.

## Abbreviations

Ang II: Angiotensin II
CAM: Cell adhesion molecule
CCA: Common carotid artery
DMEM: Dulbecco’s Modified Eagle’s Medium
ECM: Extracellular matrix
Gapdh: Glyceraldehyde 3-phosphate dehydrogenase
hAoSMC: human aortic smooth muscle cell
ICAM-1: Intercellular adhesion molecule-1
IHC: Immunohistochemistry
iNOS: inducible NO synthase
MCP-1: Monocyte chemotactic protein-1
MFG-E8: Milk fat globule–epidermal growth factor 8
MMP-2: Matrix metalloproteinase-2
MMP-9: Matrix metalloproteinase-9
NF-κB: nuclear factor-κB
PBS: Phosphate-buffered saline
PCR: Polymerase chain reaction
PDGF-BB: Platelet-derived growth factor-BB
TNF-α: tumor necrosis factor-α
VCAM-1: Vascular adhesion molecule-1
VSMC: Vascular smooth muscle cell
