# Supplementary material for: MFG-E8 mediates arterial aging by promoting the proinflammatory phenotype of vascular smooth muscle cells
Source: J Biomed Sci. 2019 Aug 30;26:61. doi: 10.1186/s12929-019-0559-0 (PMC6716880; doi:10.1186/s12929-019-0559-0)
Supplement: Supplementary file 1 — Figure S1. MFG-E8 abundantly expresses in aged human aortas and augments transactivation of NF-κB p65 in human VSMCs. (a–j) Representative IHC images display increased coexpression of MFG-E8 and phosphorylated NF-κB p65 (Ser536) as well as elevated ICAM-1 intensity in aged aortas. Arrows indicate the autofluorescence of the elastic laminae in the vessels; bar, 100 μm. (k–l) After treatment with rMFG-E8 (250 ng/mL) for 24 h, hAoSMCs were stimulated with Ang II (1 μM) for 24 h to mimic aging. (k) Cell lysates were analyzed through immunoblotting with antibodies specific for NF-κB p65 phosphorylated at Ser536 and NF-κB p65. Levels of phosphorylated p65, normalized to that of total p65 (n = 3), were analyzed. (l) The protein expression of VCAM-1 in hAoSMCs was evaluated through immunoblotting; the quantitative analysis results for VCAM-1, normalized to that of Gapdh, are displayed (n = 3). Data are presented as mean ± standard deviation. *P < 0.05 and **P < 0.01, one-way analysis of variance followed by Tukey’s multiple comparison test. (PDF 474 kb) [file 12929_2019_559_MOESM1_ESM.pdf]

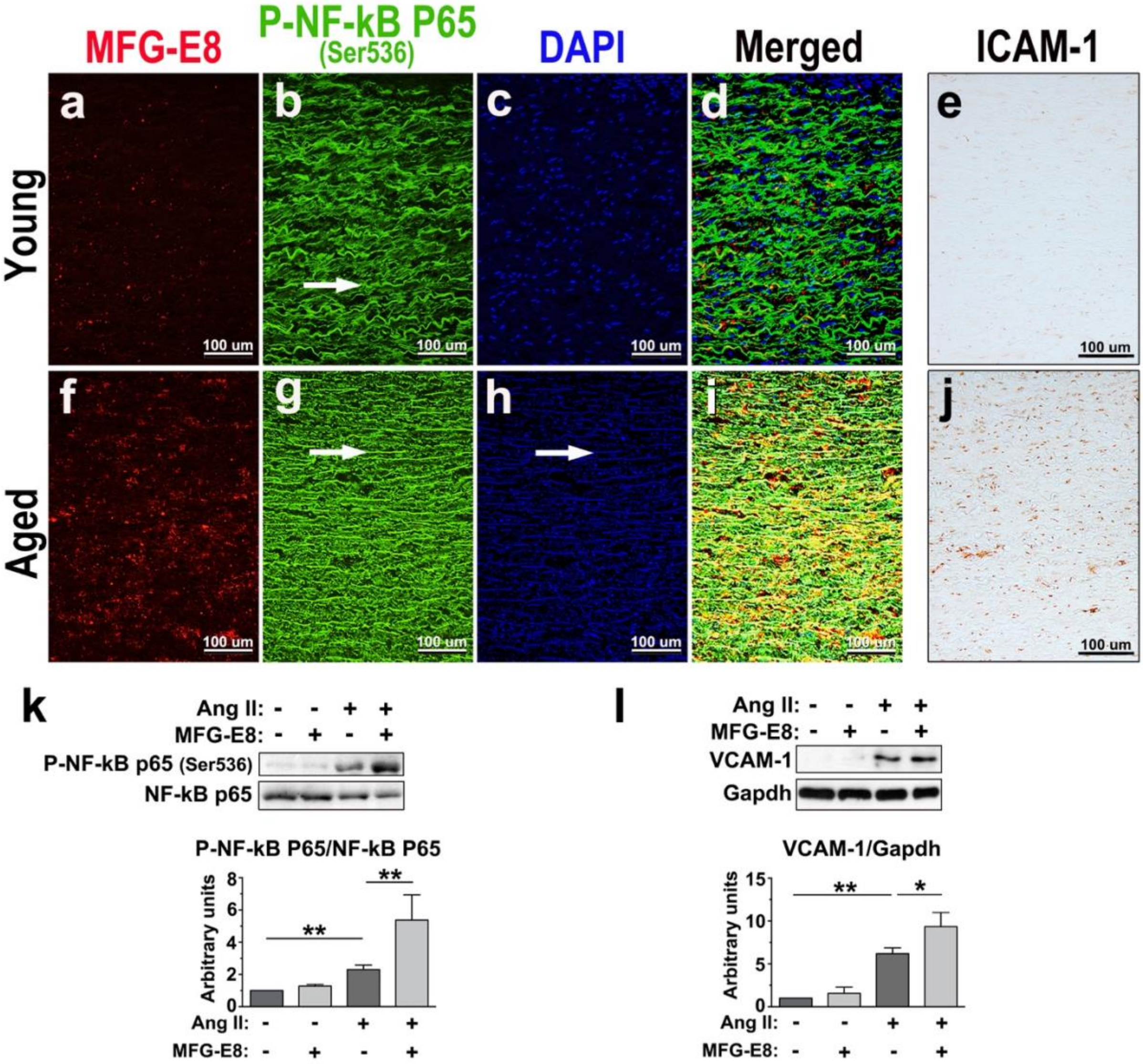

Figure S1. MFG-E8 abundantly expresses in aged human aortas and augments transactivation of NF-κB p65 in human VSMCs. (a–j) Representative IHC images display increased coexpression of MFG-E8 and phosphorylated NF-κB p65 (Ser536) as well as elevated ICAM-1 intensity in aged aortas. Arrows indicate the autofluorescence of the elastic laminae in the vessels; bar, 100 μm. (k–l) After treatment with rMFG-E8 (250 ng/mL) for 24 h, hAoSMCs were stimulated with Ang II (1 μM) for 24 h to mimic aging. (k) Cell lysates were analyzed through immunoblotting with antibodies specific for NF-κB p65 phosphorylated at Ser536 and NF-κB p65. Levels of phosphorylated p65, normalized to that of total p65 (*n* = 3), were analyzed. (l) The protein expression of VCAM-1 in hAoSMCs was

evaluated through immunoblotting; the quantitative analysis results for VCAM-1, normalized to that of Gapdh, are displayed (n = 3). Data are presented as mean ± standard deviation. *$P < 0.05$ and **$P < 0.01$, one-way analysis of variance followed by Tukey's multiple comparison test.
